# The relationship between quality of life and spiritual fortitude in individuals with an intestinal stoma

**DOI:** 10.1007/s00520-026-10876-8

**Published:** 2026-06-11

**Authors:** Asude Güney, Yadigar Ordu, Kübra Nur Köse Alabay

**Affiliations:** 1 Faculty of Nursing, Necmettin Erbakan University Meram, Konya, 42090 Yaka Mah. Beysehir Cad. D Blok No:281, Turkey; 2 Zubeyde Hanim Faculty of Health Sciences, Nigde Omer Halisdemir University, Derbent Yerleşkesi, Niğde, 51200 Atatürk Blv, Turkey

**Keywords:** Nurse, Spiritual fortitude, Ostomy, Stoma, Quality of life

## Abstract

**Purpose:**

This study aims to examine the relationship between quality of life and spiritual fortitude in individuals with an intestinal stoma.

**Methods:**

This descriptive and correlational study was conducted with 66 individuals with an intestinal stoma between November 2024 and May 2025. Data were collected face-to-face in a hospital setting using the “Descriptive Characteristics Form,” the “Stoma Quality of Life Scale,” and the “Spiritual Fortitude Scale.” Data were analyzed using SPSS 22.0, and *p* < 0.05 was considered statistically significant.

**Results:**

The participants had a mean age of 61.71 ± 13.90 years; 53% were women, and 45.5% were primary school graduates. The mean total score for Stoma Quality of Life Scale was 52.21 (SD = 8.21), and the mean total score for Spiritual Fortitude was 33.79 (SD = 8.20). There was a significant association between the spiritual fortitude of individuals with an intestinal stoma and their quality of life (*β* = 0.261, *R* = 0.261, *p* = 0.035).

**Conclusion:**

Nurses can provide spiritual counseling services to enhance the spiritual fortitude of individuals with an intestinal stoma. Future research should focus on nurses’ competencies in spiritual counseling and the effectiveness of such services.

## Introduction

Stoma is a surgical intervention performed for the treatment of gastrointestinal or urinary system cancers, inflammatory bowel diseases, and trauma [1]. Stoma creation is preferred to increase individuals’ survival rates. However, living with a stoma also brings numerous negative experiences [2, 3].

Individuals with a stoma face many problems, such as body image disturbances, difficulties participating in social activities, negative effects on work life, and disruption of sexual life [4, 5]. The physical, social, psychological, and sexual issues experienced by individuals with a stoma negatively affect their quality of life [6, 7]. Individuals may have difficulty adapting to the physical and psychosocial changes caused by the stoma [4]. Identifying methods that enable individuals with a stoma to cope effectively with changes in their lives is of great importance [2, 3].

Spiritual fortitude refers to an individual’s capacity to recover from difficulties or to overcome changes they experience successfully. This concept differs from general psychological resilience in the spiritual depth it possesses. Psychological resilience primarily refers to a recovery process. However, spiritual fortitude refers to the individual drawing the strength needed during this process directly from their own belief system or spiritual values [8, 9]. Unlike psychological resilience, spiritual fortitude is a structure of resilience that draws its strength not from the individual’s cognitive coping skills, but directly from their belief system and spiritual values [10].

Spiritual fortitude holds a significant role for individuals with chronic illnesses. It may help individuals cope with stoma-related problems, accept the stoma more quickly, improve their quality of life, adhere to treatment, become aware of their values as human beings, and adopt a more positive outlook on life [11, 12].

Spiritual fortitude, which positively supports the mental health of individuals with a stoma, may enable them to cope more effectively with challenges [4, 11, 12]. This study aimed to determine the relationship between quality of life and spiritual fortitude in individuals with an intestinal stoma.

The research questions are as follows:

1. What are the levels of spiritual fortitude in individuals with an intestinal stoma?
2. What are the levels of quality of life in individuals with an intestinal stoma?
3. What is relationship between quality of life and spiritual fortitude in individuals with an intestinal stoma?

## Methods

### Study design

This descriptive and correlational cross-sectional study was conducted between November 2024 and May 2025 with individuals with a stoma who applied to a university hospital in Türkiye.

### Study population and sample

The study population consisted of individuals with a stoma who applied to a university hospital. The sample size was calculated using G*Power (3.1.9.7) software, based on the study by Türkben Polat and Burucu [13], which examined the effect of spiritual well-being on self-efficacy in patients with a stoma. Using the correlation method for two continuous variables (Correlation: Point biserial model), with a medium effect size (*ρ* = 0.39), 95% power, and a 5% margin of error, the required sample size was calculated to be 62. Considering the possibility of data loss, data were collected from 66 participants.

### Inclusion criteria

Inclusion criteria were having undergone intestinal stoma surgery for the first time for any reason, being 18 years or older, having lived with the stoma for at least 3 months, understanding and speaking Turkish, being conscious, and volunteering to participate in the study.

### Exclusion criteria

Exclusion criteria were being in the terminal stage, having a psychiatric illness that impairs communication or perception, incomplete or incorrect completion of the data collection forms, and refusal to participate in the study.

### Data collection tools

Data were collected using the “Descriptive Characteristics Form,” the “Stoma Quality of Life Scale (SQOL),” and the “Measure of Spiritual Fortitude (SF).”

#### Descriptive characteristics form

This form, developed based on a literature review, consists of 15 closed-ended questions regarding some demographic and health-related characteristics of individuals with a stoma (e.g., age, gender, body mass index, education level, marital status, place of residence, occupation, income status, presence of social support, tobacco/alcohol use, presence of chronic/additional illness, type of stoma, duration and nature of the stoma, stoma care) [14, 15].

### Stoma Quality of Life Scale (SQOL)

The scale was developed by Baxter et al. [16] and adapted to Turkish by Karadağ et al. [17]. Excluding the first two items, the scale consists of 17 items and three sub-dimensions and is rated on a five-point Likert scale (1 = Never, 2 = Rarely, 3 = Occasionally, 4 = Frequently, 5 = Always). The sub-dimensions are as follows: work/social life (items 3–6, 18–19), sexuality/body image (items 7–9, 12, 15), and stoma function (items 10, 11, 13, 14, 16, 17). The first two items measure general satisfaction with life and are scored between 0 and 100 (0 = complete dissatisfaction, 100 = complete satisfaction). The scale uses a numerical scoring system, with each sub-dimension evaluated on a scale of 0 to 100 (0 = poor quality of life, 100 = good quality of life). The Cronbach’s alpha of the original scale was 0.87 [17], and in the present study, it was 0.908.

### Measure of Spiritual Fortitude (SF)

Developed by Van Tangoren et al. [9] and validated in Turkish by Cantaş [18], this scale consists of 9 items in three sub-dimensions and uses a five-point Likert scale (1 = Strongly disagree, 2 = Disagree, 3 = Neither agree nor disagree, 4 = Agree, 5 = Strongly agree). The sub-dimensions are spiritual fortitude (items 1, 4, 7), spiritual initiative (items 2, 5, 8), and redemptive purpose (items 3, 6, 9). The total score ranges from 9 to 45 [9]. The Cronbach’s alpha was 0.86 in the original study and 0.962 in the present study.

### Data collection

Data were collected face-to-face by the researchers at the hospital between November 2024 and May 2025. Participants were informed about the study, their consent was obtained, and they were asked to complete the data collection tools. The tools were completed in approximately 15–20 min.

## Data analysis

Data were analyzed using SPSS (version 22.0, IBM, New York, USA). Normality was tested using the Kolmogorov-Smirnov test. Descriptive data analysis included frequency, percentage, mean, standard deviation, minimum, and maximum values. The Independent Samples *T*-Test was used to compare two independent groups, while ANOVA was used for comparisons among more than two groups. Differences between groups were determined using the Tukey. Regression analysis was used to examine the relationship between the scales. A significance level of *p* < 0.05 was considered statistically significant.

### Ethical considerations

The study received written approval from both the Çankırı Karatekin University Health Sciences Ethics Committee (dated October 15, 2024; Ref No.: 16) and the relevant institution (dated November 19, 2024; Ref No.: E-14567952-900-595086). Before the study began, all participants were informed in detail about the study’s purpose, voluntary participation was ensured, and written informed consent was provided. The study was conducted in accordance with the Declaration of Helsinki.

## Results

The mean age of the participants was 61.71 ± 13.90 years. Of the participants, 53% were female, 87.9% were married, 45.5% had completed primary education, and 69.7% reported income equal to their expenditures. Additionally, 87.9% received social support from their families, 77.3% had been living with a stoma for 3–12 months, 57.6% had a permanent stoma, and 80.3% had a colostomy. It was also determined that 63.6% had a chronic or comorbid condition, and 60.6% were responsible for their own stoma care (Table 1).

**Table 1:** Participants’ descriptive characteristics (*n* = 66)

Descriptive characteristics	*n*	%
**Age (x̄ ± SD)**	61.71 ± 13.90 (Min = 25 Max = 85)	
**Gender**		
Female	35	53.0
Male	31	47.0
**Marital status**		
Married	58	87.9
Single	8	12.1
**Educational status**		
Literate	10	15.1
Elementary school	30	45.5
Middle school	15	22.7
High school	11	16.7
**Smoking status**		
Yes	28	42.4
No	38	57.6
**Monthly income status**		
Income exceeds expenses	8	12.1
Income equals expenses	46	69.7
Income is less than expenses	12	18.2
**Employment status**		
Employed	6	9.1
Unemployed	60	90.9
**Social support**		
None	6	9.1
Family	58	87.9
Friends	2	3.0
**Place of residence**		
City center (province/district)	57	86.4
Town/village	9	13.6
**BMI**		
Below 18.5	13	19.7
18.5–24.9	25	37.9
25.0–29.9	18	27.3
30.0–34.9	10	15.1
**Chronic/additional illnesses**		
Yes	42	63.6
No	24	36.4
**Stoma duration**		
3–12 months	51	77.3
13–24 months	12	18.2
25 months and over	3	4.5
**Stoma characteristics**		
Permanent	38	57.6
Temporary	28	42.4
**Stoma type**		
Colostomy	53	80.3
Ileostomy	13	19.7
**Stoma care**		
Self	40	60.6
Family member	26	39.4

*BMI* body mass index, *x̄* mean, *SD* standard deviation, *Min* minimum, *Max* maximum

The participants’ mean total score on the Stoma Quality of Life (SQOL) Scale was 52.21 ± 8.21, and their mean total score on the Spiritual Fortitude (SF) Scale was 33.79 ± 8.20. The mean scores for the SQOL subdimensions were as follows: work/social life (51.45 ± 12.67), sexuality/body image (47.65 ± 16.62), stoma function (57.51 ± 15.97), and satisfaction (58.03 ± 19.49). The mean scores for the SF subdimensions were as follows: spiritual fortitude (11.41 ± 2.89), spiritual initiative (11.26 ± 2.96), and transcendent purpose (11.12 ± 2.81) (Table 2).

**Table 2:** SQOL and SF scores (*n* = 66)

Scales and subscales	Mean ± SD	Min	Max
**Stoma quality of life scale**			
Work/social life	51.45 ± 12.67	12.50	75.00
Sexuality/body image	47.65 ± 16.62	20.00	85.00
Stoma function	57.51 ± 15.97	16.67	87.50
**Total score**	52.21 ± 8.21	26.39	70.56
**Satisfaction**	58.03 ± 19.49	10.00	100.00
**Spiritual fortitude scale**			
Spiritual fortitude	11.41 ± 2.89	5.00	15.00
Spiritual initiative	11.26 ± 2.96	3.00	15.00
Rescuer purpose	11.12 ± 2.81	4.00	15.00
**Total score**	33.79 ± 8.20	15.00	45.00

*SD* standard deviation, *Min* minimum, *Max* maximum

The mean total SQOL score of middle school graduates was found to be higher than that of literate individuals (*p* = 0.014). The mean total SQOL score of individuals residing in urban centers surpassed that of those inhabiting rural areas (*p* = 0.010). The mean SQOL total score of individuals with a BMI between 30.0 and 34.9 was higher than that of individuals with a BMI between 18.5 and 24.9 and between 25.0 and 29.9 (*p* = 0.001). The mean SQOL total score of individuals with permanent stomas was found to be higher than that of those with temporary stomas (*p* = 0.031). The mean total SQOL score of those who performed stoma care themselves was higher than that of those who had stoma care performed by a caregiver (*p* = 0.036).

Women had significantly higher total SF scores compared to men (*p* = 0.036). Similarly, participants with income equal to their expenses had significantly higher SF scores than those with insufficient income (*p* = 0.023). Those receiving social support from family had significantly higher SF scores compared to those who received support from friends (*p* = 0.006). Participants living in urban areas had significantly higher SF scores than those residing in rural areas (*p* = 0.024). Additionally, those with permanent stomas had significantly higher SF scores than those with temporary stomas (*p* = 0.007) (Table 3).

**Table 3:** Comparison of descriptive characteristics with SQOL and SF scores (*n* = 66)

Descriptive characteristics	SQOL		SF	
	Total score		Total score	
	Mean ± SD	Min–Max	Mean ± SD	Min–Max
**Gender**				
Female	52.31 ± 5.18	40.56–64.44	35.77 ± 7.33	16.00–45.00
Male	52.09 ± 10.76	26.39–70.56	31.55 ± 8.66	15.00–45.00
**t/p**	0.105/0.917		**2.145/0.036**	
**Marital status**				
Married	52.62 ± 8.21	26.39–70.56	34.41 ± 8.23	15.00–45.00
Single	49.20 ± 8.13	40.00–67.22	29.25 ± 6.78	18.00–36.00
**t/p**	1.105/0.273		1.693/0.095	
**Education level**				
Literate^a^	47.06 ± 10.25	26.39–57.50	32.70 ± 6.52	23.00–40.00
Elementary school^b^	53.72 ± 6.24	42.78–66.94	35.63 ± 8.14	16.00–45.00
Middle school^c^	55.57 ± 7.36	43.61–70.56	32.53 ± 9.13	15.00–42.00
High school^d^	48.16 ± 9.30	26.39–58.89	31.45 ± 8.42	18.00–45.00
**F/p**	3.824/0.014, c > a*		0.978/0.409	
**Smoking status**				
Yes	53.07 ± 7.67	40.00–67.22	31.50 ± 8.34	15.00–43.00
No	51.57 ± 8.64	26.39–70.56	35.47 ± 7.78	16.00–45.00
**t/p**	0.728/0.470		−1.989/0.051	
**Monthly income status**				
Income exceeds expenses ^a^	51.35 ± 7.45	40.00–58.89	31.75 ± 8.78	18.00–45.00
Income equals expenses ^b^	53.07 ± 7.19	26.39–70.56	35.50 ± 7.59	15.00–45.00
Income less than expenses ^c^	49.44 ± 11.84	26.39–67.22	28.58 ± 8.26	16.00–45.00
**F/p**	0.977/0.382		4.003/0.023, b > c*	
**Employment status**				
Employed	49.49 ± 6.86	40.00–56.11	29.00 ± 6.57	18.00–38.00
Not employed	52.48 ± 8.34	26.39–70.56	34.27 ± 8.24	15.00–45.00
**t/p**	−0.847/0.400		−1.514/0.135	
**Social support**				
None ^a^	53.10 ± 7.00	48.61–67.22	28.17 ± 8.64	16.00–36.00
Family ^b^	52.39 ± 8.35	26.39–70.56	34.86 ± 7.64	15.00–45.00
Friends ^c^	44.17 ± 5.89	40.00–48.33	19.50 ± 2.12	18.00–21.00
**F/p**	1.008/0.371		5.647/0.006, b > c*	
**Place of residence**				
City center (province/district)	53.23 ± 7.78	26.39–70.56	34.68 ± 7.93	15.00–45.00
Town/village	45.71 ± 8.32	26.39–52.50	28.11 ± 8.02	16.00–41.00
**t/p**	**2.670/0.010**		**2.307/0.024**	
**BMI**				
Below 18.5^a^	52.74 ± 6.25	42.78–64.44	33.92 ± 9.98	15.00–45.00
18.5–24.9^b^	48.71 ± 9.20	26.39–60.00	33.36 ± 8.10	18.00–45.00
25.0–29.9^c^	52.05 ± 5.90	40.56–67.22	32.39 ± 8.42	16.00–45.00
30.0–34.9^d^	60.53 ± 5.69	52.22–70.56	37.20 ± 5.16	26.00–43.00
**F/p**	6.120/0.001, d > c > b*		0.767/0.517	
**Chronic/additional illness**				
Yes	52.84 ± 8.37	26.39–70.56	33.31 ± 8.53	15.00–45.00
No	51.10 ± 7.99	26.39–67.22	34.63 ± 7.71	18.00–45.00
**t/p**	0.825/0.413		−0.624/0.535	
**Stoma duration**				
3–12 months	52.24 ± 8.06	26.39–67.22	33.55 ± 8.49	15.00–45.00
13–24 months	50.14 ± 8.06	35.00–60.00	33.17 ± 7.47	22.00–45.00
25 months and above	59.91 ± 9.76	51.39–70.56	40.33 ± 3.06	37.00–43.00
**F/p**	1.738/0.184		1.012/0.369	
**Stoma characteristics**				
Permanent	54.06 ± 7.60	35.00–70.56	36.21 ± 6.56	21.00–45.00
Temporary	49.68 ± 8.47	26.39–61.94	30.50 ± 9.14	15.00–43.00
**t/p**	**2.205/0.031**		**2.815/0.007**	
**Stoma type**				
Colostomy	51.88 ± 8.30	26.39–67.22	34.19 ± 7.53	16.00–45.00
Ileostomy	53.53 ± 8.02	40.56–70.56	32.15 ± 10.72	15.00–45.00
**t/p**	−0.644/0.522		0.646/0.528	
**Stoma care**				
Self	53.90 ± 6.68	40.00–67.22	34.05 ± 8.85	15.00–45.00
Caregiver	49.59 ± 9.71	26.39–70.56	33.38 ± 7.25	19.00–44.00
**t/p**	**2.139/0.036**		0.320/0.750	

Significant results are shown in bold

*SD* standard deviation, *Min* minimum, *Max* maximum, *t* Independent Sample *T*-Test, *F* ANOVA

*Tukey

The relationship between SF and SQOL total scores was examined using linear regression analysis. The regression model was statistically significant (*F* = 4.665, *p* = 0.035), and the model explained 5.3% of the variance in SQOL scores (Adjusted *R*^2^ = 0.053; *p* = 0.035). A one-point increase in spiritual fortitude led to an increase of 0.261 points in the total SQOL score (SE = 0.121; *p* = 0.035). This finding indicates that spiritual fortitude enhances the quality of life among individuals with intestinal stomas (Table 4).

**Table 4:** The effect of mental fortitude on the quality of life of individuals with intestinal stoma (*n* = 66)

Scale	*β*_0_ (%95 CI)	SH	*β*_1_	*t*	*p*	*r*^1^	*r*^2^	VIF
Constant	43.386	4.200		10.330	< 0.001			
Spiritual fortitude	0.261	0.121	0.261	2.160	0.035	0.261	0.261	1.000

*F* = 4.665, *p* = 0.035, *R*^2^ = 0.068, Adjusted *R*^2^ = 0.053, *β*_0_: Unstandardized beta coefficient, *β*_1_: Standardized beta coefficient, *r*^1^: Zero-order correlation, *r*^2^: Partial correlation

## Discussion

A stoma can negatively affect individuals’ quality of life in physical, social, spiritual, and psychological domains. Spiritual fortitude serves as an important strength in coping with the adverse effects caused by a stoma. To the best of our knowledge, this is the first study to investigate the relationship between spiritual fortitude and quality of life in individuals with an intestinal stoma.

According to our findings, the mean total SQOL score among individuals with an intestinal stoma was at a moderate level, while the mean total SF score was above average. Previous studies have reported that the quality of life among individuals with a stoma tends to be moderate [6, 19–22] or low [23–25]. Self-assessed spirituality [26] and spiritual well-being [13], on the other hand, were found to be above average. Sociodemographic and clinical characteristics play a significant role in the variation of SQOL and SF scores [20, 21, 26]. In this study, variables such as education level, income, presence of chronic/comorbid conditions, social support, type and characteristics of the stoma, duration, and stoma care may have contributed to differences in SQOL and SF scores.

This study primarily aimed to assess the relationship between spiritual fortitude and quality of life. In line with this overall objective, our findings were supported by sociodemographic data. In this study, middle school graduates with a high BMI and those who manage their own stoma care were found to have higher average quality of life scores. A body of research has indicated a direct correlation between educational attainment and access to cancer prevention strategies. According to the findings of Leite Junior et al. (2025) [20], an inadequate level of health literacy has been demonstrated to exert a detrimental influence on the quality of life of individuals by impeding the capacity to proactively avert the onset of maladies. The present study posits that the heightened educational attainment of middle school graduates relative to literate individuals may have contributed to the observed enhancement in quality of life. A body mass index (BMI) that is lower than the accepted healthy range has been reported to have a deleterious effect on the psychological well-being of individuals with stomas, and consequently, on their quality of life. However, contrary to this research, a study revealed that individuals with a normal BMI exhibited higher psychological well-being compared to those with underweight, overweight, and obese classifications [27]. Conversely, another study found that patients with colon cancer and a high BMI had a low health-related quality of life [28]. The present study posits that the correlation between weight reduction and disease progression, as well as the association between obesity and physical fortitude, may have influenced the enhancement in quality of life among individuals with elevated BMI. A direct relationship between self-care and quality of life has been reported in individuals with stomas. A study revealed that inadequate self-care in stoma patients may result in diminished health-related quality of life [29]. The fact that the majority of patients manage their stoma care independently may have contributed positively to their quality of life by enhancing their sense of self-efficacy and control. The present study posits that individuals who engaged in self-managed stoma care, accepted their stoma, and received support for self-care and independence may have contributed to an enhanced quality of life.

In addition, higher SF scores were found among women, individuals whose income matched their expenses, those with family-based social support, those living in urban areas, and those with permanent stomas. Studies have also shown that women tend to seek social support and participate more actively in self-care practices, which positively affects their perception of physical well-being [20, 30]. Furthermore, individuals with permanent stomas are reported to adapt better over time [23]. The higher SF scores found in our study may be attributed to the fact that the sample predominantly consisted of women (who tend to perceive better well-being), the presence of family support, improved access to healthcare services in urban areas, and better adaptation to permanent stomas. Even in the early stages, the presence of high levels of spiritual fortitude may stem from the fact that individuals’ processes of making sense of their own experiences, coping strategies rooted in religious beliefs, and strong family support facilitate the adjustment process.

This study found a significant association between the spiritual fortitude and quality of life of individuals with an intestinal stoma. Supporting our findings, previous research has also identified a significant relationship between quality of life and spiritual well-being in patients with a stoma [31]. Spiritual fortitude helps individuals cope with stress and trauma by drawing on their spiritual beliefs and values [32]. Religion and spirituality play important roles in promoting individual well-being, coping with life challenges, treatment acceptance, and post-treatment lifestyle changes [33]. Religious practices such as prayer and fasting, along with behaviors like patience and surrender, have been found to enhance life satisfaction and strengthen spiritual fortitude [32]. Since the participants in this study were all Muslims, the fulfillment of religious practices and the strong connection between religion and spirituality may have contributed to their higher levels of spiritual fortitude and, consequently, improved quality of life. The low level of explanatory power of the relationship between variables demonstrates that quality of life is a complex construct shaped by the interaction of multiple psychosocial and clinical factors.

## Limitations

This study has some limitations regarding its generalizability. Since all participants were Muslim individuals in Turkey, cultural and religious values such as patience and resignation may have influenced their levels of spiritual fortitude. This may limit the generalizability of the findings to secular or non-Muslim societies. The fact that the sample consists largely of married individuals with family support may have led to insufficient representation of individuals with limited social support, which may have limited the generalizability of the results. The sample of this study consisted solely of participants from a single hospital. Therefore, the results are specific to this sample and cannot be generalized to the broader population.

## Conclusion

The total SQOL score of individuals with intestinal stomas was found to be moderate, while the total SF score was above average. The mean SQOL score was found to be higher among individuals who had graduated from middle school, resided in the city center, had a high BMI, had a permanent stoma, and performed their own stoma care. Higher SF scores were observed among women, individuals whose income matched their expenses, those receiving family support, those living in city centers, and individuals with permanent stomas. Spiritual fortitude improved the quality of life among these individuals, and a positive relationship was identified between the two. It is recommended that nurses provide spiritual guidance services to support the development of spiritual fortitude in individuals with a stoma. It is recommended that nurses possess the awareness necessary to identify patients’ spiritual care needs and effectively utilize their collaborative roles to facilitate communication with the hospital’s spiritual support unit on behalf of individuals who require such support. To ensure the effective implementation of this process, competency-based in-service training programs should be organized for nurses; these programs should focus on identifying spiritual needs, establishing a trust-based relationship with the patient, effective communication, and developing a holistic care approach. Future research should focus on nurses’ competencies in spiritual guidance and the effectiveness of such services. In future studies, it is recommended that the effects of individual and clinical characteristics-such as the reason for stoma creation and whether the procedure was planned or performed as an emergency-on spiritual fortitude be comprehensively examined.

## Data Availability

The data that support the findings of this study are not openly available due to reasons of sensitivity and are available from the corresponding author upon reasonable request.
